# Metabolic pathways that permit *Mycobacterium avium* subsp. *hominissuis* to transition to different environments encountered within the host during infection

**DOI:** 10.3389/fcimb.2023.1092317

**Published:** 2023-04-14

**Authors:** Norah Abukhalid, Rajoana Rojony, Lia Danelishvili, Luiz E. Bermudez

**Affiliations:** ^1^ Department of Biomedical Sciences, College of Veterinary Medicine, Oregon State University, Corvallis, OR, United States; ^2^ College of Applied Medical Sciences, King Saud bin Abdulaziz University for Health Sciences, Riyadh, Saudi Arabia; ^3^ King Abdullah International Medical Research Center, Riyadh, Saudi Arabia; ^4^ Department of Microbiology, College of Science, Oregon State University, Corvallis, OR, United States

**Keywords:** M. avium, proteomics, aerobic, anaerobic, biofilm, stress conditions, metabolic pathways

## Abstract

**Introduction:**

*M. avium subsp. hominissuis (M. avium)* is an intracellular, facultative bacterium known to colonize and infect the human host through ingestion or respiratory inhalation. The majority of pulmonary infections occur in association with pre- existing lung diseases, such as bronchiectasis, cystic fibrosis, or chronic obstructive pulmonary disease. *M. avium* is also acquired by the gastrointestinal route in immunocompromised individuals such as human immunodeficiency virus HIV-1 patients leading to disseminated disease. A hallmark of *M. avium* pulmonary infections is the ability of pathogen to form biofilms. In addition, M. avium can reside within granulomas of low oxygen and limited nutrient conditions while establishing a persistent niche through metabolic adaptations.

**Methods:**

Bacterial metabolic pathways used by *M. avium* within the host environment, however, are poorly understood. In this study, we analyzed *M. avium* proteome with a focus on core metabolic pathways expressed in the anaerobic, biofilm and aerobic conditions and that can be used by the pathogen to transition from one environment to another.

**Results:**

Overall, 3,715 common proteins were identified between all studied conditions and proteins with increased synthesis over the of the level of expression in aerobic condition were selected for analysis of in specific metabolic pathways. The data obtained from the *M. avium* proteome of biofilm phenotype demonstrates in enrichment of metabolic pathways involved in the fatty acid metabolism and biosynthesis of aromatic amino acid and cofactors. Here, we also highlight the importance of chloroalkene degradation pathway and anaerobic fermentationthat enhance during the transition of *M. avium* from aerobic to anaerobic condition. It was also found that the production of fumarate and succinate by MAV_0927, a conserved hypothetical protein, is essential for M. avium survival and for withstanding the stress condition in biofilm. In addition, the participation of regulatory genes/proteins such as the TetR family MAV_5151 appear to be necessary for *M. avium* survival under biofilm and anaerobic conditions.

**Conclusion:**

Collectively, our data reveal important core metabolic pathways that *M. avium* utilize under different stress conditions that allow the pathogen to survive in diverse host environments.

## Introduction

*Mycobacterium avium* subspecies *hominissuis* (*M. avium*) is a member of the *Mycobacterium avium* complex, which also includes *M. avium* subsp. *avium*, *M. avium* subsp*. paratuberculosis*, *Mycobacterium chimaera* and *Mycobacterium intracellulare* (Zheng and Fanta, 2013; Koh, 2017). *M. avium* is an opportunistic pathogen causing pulmonary infections in individuals with chronic lung conditions such as chronic obstructive pulmonary disease and cystic fibrosis (Koh, 2017) and disseminated infections in immunocompromised patients such as patients with HIV/AIDS and or people with genetic mutations in genes involved in immune defenses (Piersimoni and Scarparo, 2009). The overall prevalence of *M. avium* infection in US is estimated to be between 1.4 and 13.9 per 100,000 persons. The infection incidence is increasing by 2.5-8% annually and varies by region, sex, and race/ethnicity (Adjemian et al., 2018).

During initial colonization of the lung airways, *M. avium* forms microaggregates that is composed of 3 to 20 bacteria of pre-biofilm phenotypic state (Babrak et al., 2015b), allowing the pathogen to efficiently colonize mucosal surfaces (Babrak et al., 2015b). The mucus is composed of several immune defense factors including glycoproteins (mucins), digestive enzymes, antimicrobial peptides, and immunoglobulins (Lillehoj et al., 2013) that are involved in binding and removal of microorganisms from the lung airways. However, the microaggregate binding protein (MBP-1) and microaggregate invasion protein (MIP-1) of *M. avium* were implicated in the modulation of inflammatory responses at the mucosal surface and promoting efficient uptake of bacteria by epithelial cells (Sangari et al., 2001; Babrak et al., 2015a). Later, *M. avium* escape and spread from the epithelial layer to other sites of the lungs forming more lesions (McGarvey and Bermudez, 2002). Within the host, the pathogen transits through varied environments and, depending on the site of infection, it can reside either within the phagosome vacuoles of epithelial cells or macrophages, form biofilms in the respiratory mucosa or live in structures known as granulomas and nodes (McNabe et al., 2011).

The metabolism plays a central role in initiation and maintenance of tolerance mechanisms in bacteria as well as in reactivation and in transitioning the non-replicating to actively growing state. In the intracellular or extracellular milieu of the host, *M. avium* encounters the low oxygen tension, increased osmolarity and pH, and nutrient deprived conditions, stimulating phenotypic changes such as the low metabolic and low growth rates. These alterations allow the pathogen to tolerate diverse stress conditions, resist killing by host defenses and the action of antibiotics (Maurer et al., 2014; Oh et al., 2014; Danelishvili et al., 2020; Rojony et al., 2020) while establishing survival niche in different environmental conditions. The underlying mechanisms of *M. avium* metabolic alterations during infection are multiple (McNabe et al., 2011). Recent studies suggest that upregulation of damage/repair functions such as oxidative stress and reactive oxygen species (ROS) can stimulate bacterial efflux system for recycling of damaged proteins and enhance mycobacterial tolerance mechanism (Park et al., 2003). In addition, *M. avium* has an ability to grow as a sessile, three-dimensionally organized and multicellular communities called biofilms. The limitation of nutrients and the lack of oxygen within the biofilm matrix induce a nonreplicating state in bacteria as well as promote metabolic heterogeneity, which is characterized with a wide range of physiological states (Carter et al., 2003; Archuleta et al., 2005; Rojony et al., 2019). These metabolic changes play an important role in promoting persistent phenotypes within *M. avium* biofilms (Archuleta et al., 2005).

**Figure 1:**
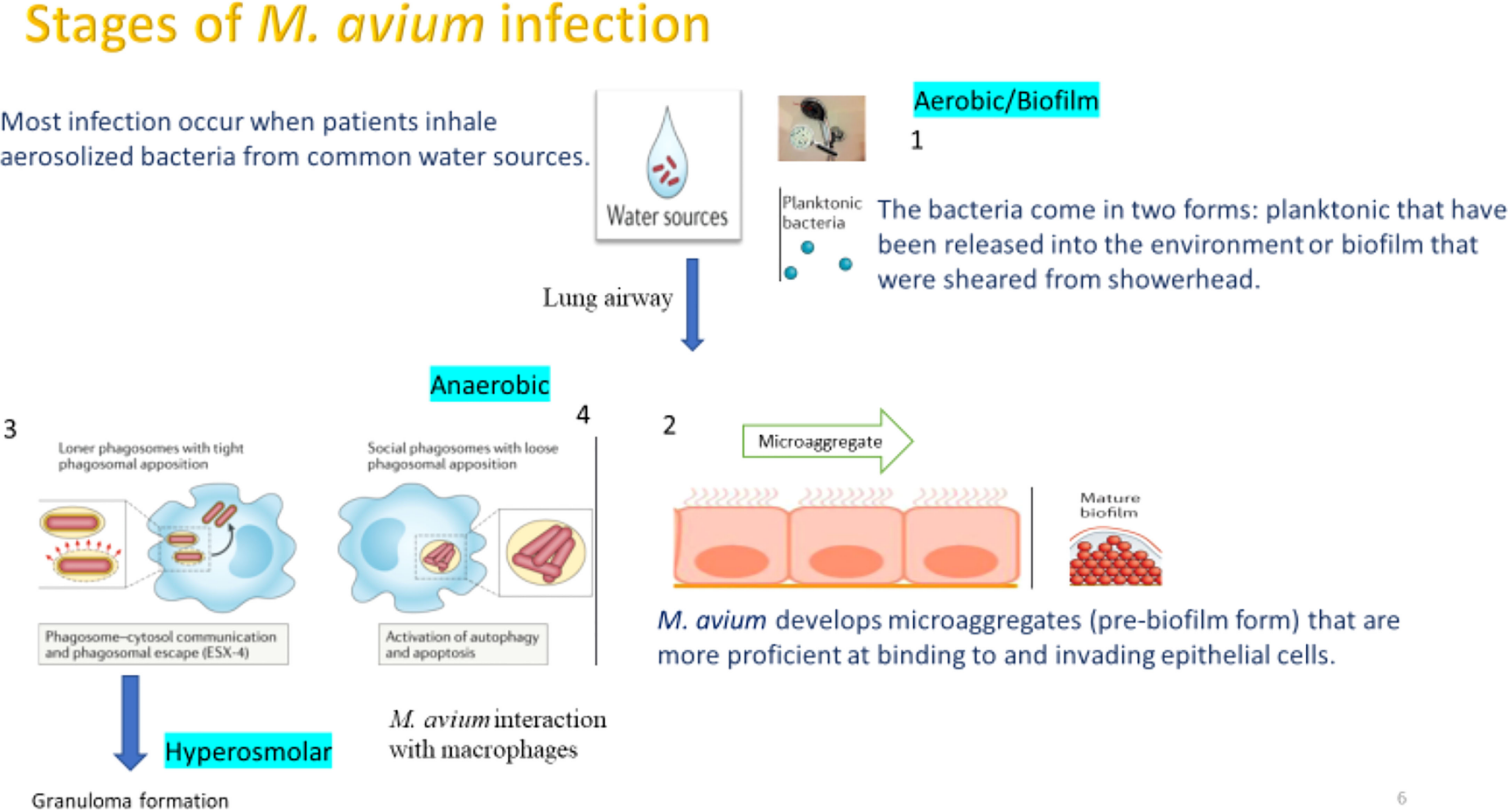
Main stages and phenotypes of *M.avium* causing a lung airway infection. The first stage *M. avium* reaches the airways in planktonic or biofilm forms. We established a model to understand the stages that both planktonic and biofilm phenotypes encounter during the infection. A key assumption of the model is that the pathogen that takes a long time to synthesize proteins, does need a “core pathways” to keep the metabolism active and by connecting with pathways exclusive to the different phenotypes allow for transitions needed to synthesize proteins required for the biofilm as well as the intracellular phenotypes.

The question on how *M. avium*, an organism that is slow in the synthesis of new proteins, can adapt quickly to new environment (biofilm, intracellular, anaerobic) is a fundamental in understanding the pathogenicity mechanisms that bacteria employ in the host (**Figure 1**). Our hypothesis is that mycobacteria maintain active only necessary “core” pathways to be viable in various stress condition, and then these common metabolic pathways serve as a base that feed into environment specific pathways that are exclusive to different conditions. To identify the metabolic dynamics employed by the planktonic (aerobic and anaerobic) and biofilm forming bacteria, we performed the quantitative analysis of *M. avium* proteome under different environmental conditions and identified core pathways expressed in all tested conditions that connected to specific metabolic activities necessary for the pathogen to adapt to different environments. (Abukhalid et al., 2021)

## Materials and methods

### Bacteria and culture condition

*M. avium* strain 104 was originally isolated from the blood of an AIDS patient and has been shown to infect mice through the respiratory route (Blanchard et al., 2018). The strain104 was strain was obtained from the ATCC collection. *M. avium* was grown till mid-log phase growth (5-6 days) on Middlebrook 7H10 agar plates containing 10% oleic acid, albumin, dextrose, and catalase (OADC; Hardy Diagnostics, Santa Maria, CA) at 37°C (Rojony et al., 2019). Bacterial inoculants were prepared in Hanks’ Balanced Salt Solution (HBSS; VWR, Visalia, CA, USA), and visually adjusted to a McFarland 0.5 standard equivalent to 1.5 x 10^8^ colony forming unit (CFU)/mL cell density. The exact concentrations were determined by serially diluting bacterial samples, and quantifying CFUs on 7H10 agar plates after 7 days of incubation at 37°C. For the aerobic condition, tubes were kept in the shaking incubator at 37°C for 24 h and for the anaerobic condition samples were placed into anaerobic jar with methylene blue (redox indicator) strips as a positive control for anerobic conditions at 37°C for 24 h. Biofilms were formed for 7 days in 10 ml HBSS using 1 × 10^8^ cells/ml inoculum in the 25 cm^2^ tissue culture flasks as reported (Rojony et al., 2019; Rojony et al., 2020). Bacteria were centrifuged at 3,500 rpm for 20 min at 4°C, washed once with HBSS and lysed in 3% SDS containing EDTA-free Protease Inhibitor Cocktail (Sigma-Aldrich) with bead-beating method.

### Biofilm formation and quantification

A turbid suspension of *M. avium* was generated to obtain 3 x 10^8^ CFUs/mL as previously described (Babrak et al., 2015b; Babrak et al., 2015a). Bacteria were diluted to achieve 10^7^ CFUs/mL. The initial suspension was serial diluted and plated to elucidate the starting number of bacteria. The bacteria were aliquoted into a 96-well plate, with a volume of 150 µL per well (Grenier Bio). Biofilms were formed for either 7 or 14 days, unless otherwise stated, in the dark, at 25°C, and then quantified. Crystal violet stained the extracellular matrix of the biofilm for quantification, with no pre-washes as described (Babrak et al., 2015b; Babrak et al., 2015a). Absorbance measurements were taken on the Epoch (Biotex) using manufacturer’s software for analysis. When relevant to the experiment, biofilms were enumerated for CFUs/well by removing the supernatant, adding 200 μL of PBS-Tween to each well, and pipetting up and down at least 50 times to disrupt the biofilm. The disrupted biofilms were then serial diluted and plated on 7H10 media. The viability of the bacterial population was determined following dispersion of the biofilm, using the live-dead assay as previous reported (Babrak et al., 2015b; Babrak et al., 2015a).

### Protein sample preparations

The protocol was described previously (Rojony et al., 2019). Briefly, after culturing the bacteria at the different conditions (mentioned above), the lysates were cleared through microcentrifugation at 15,000 rpm for 10 min followed with filtration using the 0.22 µm syringe filters. Total protein concentrations were determined on a NanoDrop machine (Thermo Scientific).

### Tandem mass tag (TMT)-based mass spectrometry (MS) and data processing

The *M. avium* proteomic data is available on the ProteomeXchange through MassIVE and can be found under the identifier PXD018956. The total protein sample labeling and the quantitative mass spectrometry analysis have been previously performed and are detailed in two studies (Rojony et al., 2019; Rojony et al., 2020).

Raw data files were processed using Proteome Discoverer 2.1 with SEQUEST-HT and previously-described methods with modifications as appropriate (Entus et al., 2002; Pradeep et al., 2006; Hamilton et al., 2009; Itzert et al., 2019). MS data was searched against *M. avium* 104 reference strain (Jeffrey et al., 2017). The digesting enzyme was specified as trypsin. Only up to two missed cleavages were allowed and peptides of fewer than 6 amino acids or more than 144 amino acids were excluded. TMT relative abundance values were normalized to the pooled internal standard divided by the median of all internal standard values. The resultant values were then normalized to median signal to noise values for each label divided by the median of all channel median values to account for variable labeling efficiencies. The data was processed in the National Center for Biotechnology (NCBI) web server and was used for conserved protein domain searches. The NCBI Basic Local Alignment Search Tool (BLAST) was utilized for protein alignment analysis. Proteins with an identification confidence of 95% (p<0.05) were reported.

### Effect of the inhibition of pathways

To determine whether the inhibition of pathways could have an effect on the ability of *M.avium* to adapt to environmental conditions, we used isoniazid at 50% sub-inhibitory concentration for *M.avium* (8 μg/ml) and Triclosan at sub-inhibitory concentration (6 μg/ml). Bacteria were exposed for 5 min or 30 min to the compounds and them allow to form biofilms for 7 days. Triclosan binds to enoy-acyl carrier and prevents fatty-acid biosynthesis. Isoniazid inhibits mycolic acid synthesis (Fatty-Acid synthesis II pathway).

After 7 days, bacterial CFU/ml and biofilm mass were determined.

### Analysis of identified proteins

The relative abundance of proteins identified during different growth conditions (anaerobic and biofilm) was compared with the levels of corresponding protein found in standard growth condition (aerobic) to calculate the fold change (FC). The ratio of the changes between the growth conditions and the aerobic condition was used to compute the FC. The FC changes of protein levels from 0.5-fold downregulation to 1.5-fold or higher upregulation in comparison to aerobic are presented. Comparison between different growth conditions and aerobic conditions was carried out using ANOVA and the independent student’s t-test when appropriate. To determine the statistical assumption of equal variance between the studied conditions required for the student’s *t* test, f- test was conducted. The f test for anaerobic\biofilm variance in comparison to aerobic variance was calculated. If the variances were not equal, then the student’s *t* test with Welch’s correction was used. In both cases, A *p* value of <0.05 with two-tailed testing was considered statistically significant. Further, differences between different growth conditions and aerobic conditions were considered significant using pi-score that combines the *P* value and fold change corresponding to <0.05 (pi score > 1.1082).

## Results and discussion

### Metabolic pathways of *M. avium* activated in all tested environmental conditions of the host

**Figure 2:**
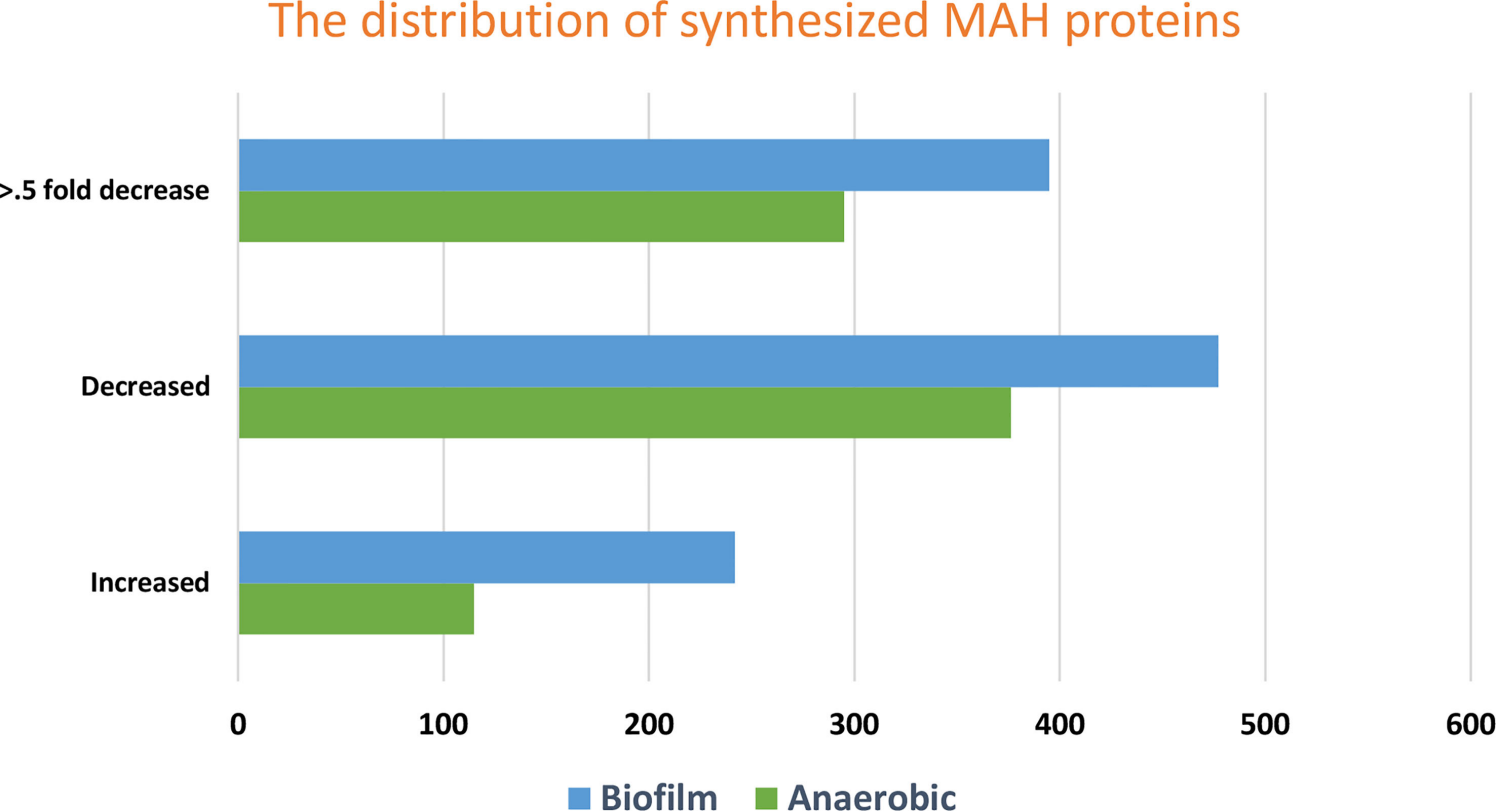
The figure shows the distribution of fold changes of proteins enriched and repressed under anaerobic and biofilm conditions when compared with the aerobic control. While the incubation under anaerobic and biofilm conditions resulted in enrichment of 115 and 242 proteins, respectively, in comparison to aerobic conditions, the synthesis of 376 and 477 proteins were downregulated when compared to *M. avium* on controlled aerobic condition. More specifically, The FC changes of protein levels of 0.5-fold downregulation in comparison to aerobic are presented. Synthesized proteins corresponding to p < 0.05 and pi score > 1.1082 were considered significant.

In response to changing environments and stress conditions of the host during bacterial infection, *M. avium* requires a well-balanced adaptation of its metabolism. Therefore, main questions that remain are (i) which metabolic pathways remain active (while others disappear) that allow bacteria to adapt the host environment? (ii) are there metabolic pathways that remain common in all stress conditions? and (iii) if these pathways may work as transition pathways? The global overview of induced and repressed *M. avium* proteome of anaerobic, biofilm and aerobic phenotypes were investigated using quantitative TMT-based mass spectrometry after 24 h exposure to the tested conditions (Rojony et al., 2019; Danelishvili et al., 2020; Rojony et al., 2020). Overall, 3,715 proteins were identified out of 5,313 to be expressed in all conditions. More specifically, while the incubation under anaerobic and biofilm conditions resulted in enrichment of 115 and 242 proteins, respectively, in comparison to aerobic conditions, the synthesis of 376 and 477 proteins were downregulated when compared to *M. avium* on controlled aerobic condition (**Figure 2**). Differences between experimental and control groups were considered significant using pi-score that combines the *P* value and fold change corresponding to <0.05 (pi score > 1.1082) (Xiao et al., 2014). The supplemental data displays a list of overall proteins identified across all groups with their normalized abundance, annotations, and fold changes over controls for each environmental condition.

**Figure 3:**
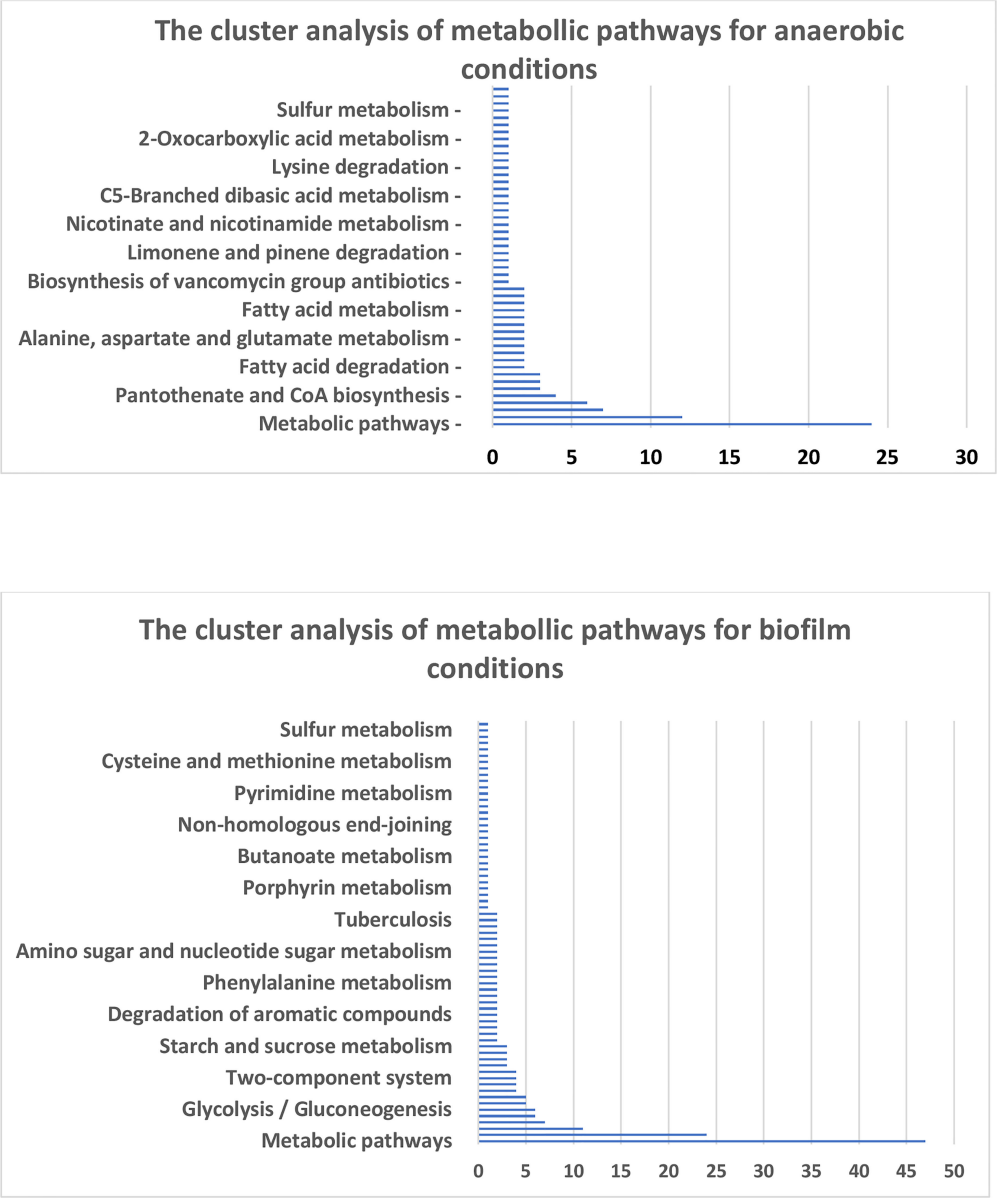
Charts represent the KEGG enrichment analysis of metabolic pathways for the corresponding conditions.

The proteins enriched in anaerobic and biofilm conditions were classified based on the metabolic pathways of the KEGG (Kyoto Encyclopedia of Genes and Genomes) database. Charts of the **Figure 3** demonstrate metabolic pathways of highly synthesized proteins in anaerobic and biofilm conditions, respectively. In anaerobic condition, the detailed cluster of the synthesized proteins belong to metabolic pathways, biosynthesis of secondary metabolite, microbial metabolism in diverse environment, glyoxylate and dicarboxylate metabolism, pyruvate metabolism and carbon metabolism (**Figure 3**). On the other hand, the detailed cluster of proteins in biofilm condition belong to metabolic pathways, biosynthesis of secondary metabolite, microbial metabolism in diverse environment, glycerolipid metabolism, carbon metabolism, biosynthesis of amino acid and biosynthesis of cofactors (**Figure 3**).

To establish changes during the adaptation of M. avium from aerobic to anaerobic/biofilm conditions, we also analyzed pathways where synthesis of metabolic enzymes was repressed during this transition. Hypoxia and biofilm conditions led to significant downregulation of M. avium factors involved in ribosomal biogenesis, suggesting an overall reduction in protein synthesis during oxygen depletion and, subsequently, a reduced growth rate (**Supplementary Table 1**). The reduction of twenty-five proteins involved in glycerolipid metabolism suggest a sharp decrease in cell wall biosynthesis, which is in anaerobic conditions was not unexpected, since in the absence of an electron acceptor, the ability to regenerate ATP and to reduce a power *via* oxidative phosphorylation were restricted and, as a result, leading to growth arrest (**Supplementary Table 1**). Also, the reduction of MAV_0238 and other ABC transporter is an indicative of M. avium disability to keep nutrient uptake and to maintain cellular functions. Therefore, under anaerobic and biofilm metabolic conditions, decreasing the rate of protein synthesis, nutrient uptake and cell wall biosynthesis and is likely beneficial for energy conservation.

Numerous sets of *de novo* synthesized proteins were found commonly expressed in all conditions and suggested that *M. avium* utilizes not only glucose as energy and carbon sources, but also amino acids, fatty acid and glycerolipids (**Figure 3**). The analyses also revealed that *M. avium* activate pathways of amino acid catabolism, specifically, branched chain amino acid, tryptophan, lysine, and histidine. In this analysis for the first time was discovered that *M. avium* potentially utilize starch or glycogen through MAV_3210/glgX glycogen debranching enzyme, in all conditions, however it is not present in a particular metabolic pathway. The MAV_3210 enzyme is responsible for glycogen- and starch-degrading activities, suggesting that *M. avium* may process glycogen during the transition between different environments. In addition, glycerol can be also used by *M. avium* as carbon source through 25 enzymes of the glycerolipid pathway identified in all conditions. Taken this data together suggest that the metabolic switch of *M. avium* in different environmental conditions is also reflected by a shift from amino acids usage to glycerolipids and fatty acid when entering anaerobic and biofilm conditions, respectively.

The acquisition of nutrients within the host is an indispensable prerequisite for *M. avium* growth and for a successful adaptation. The *M. avium* proteome enrichment with 48 various ABC transporters in different environments of the host (**Supplementary Data**) suggests how *M. avium* may be exploiting the host nutrients to support its growth and survival.

The metabolic pathways of M. avium biofilms are enriched with enzymes associated with fatty acid metabolism, amino acid and biosynthesis of cofactors. Within the environment of biofilm, microorganisms are immobilized in a self-produced matrix and in relative proximity to one another (Vestby et al., 2020). Unlike many bacterial pathogens, mycobacteria biofilms do not contain exopolysaccharides (Flemming and Wingender, 2010). The biofilm matrix components of mycobacteria include, free mycolic acids (Ojha et al., 2005; Ojha et al., 2008), extracellular DNA (eDNA) (Rose and Bermudez, 2016) and glycopeptidolipids that are part of the outermost layer of the cell wall (Freeman et al., 2006). However, if small-molecule metabolites and activation of associated metabolic pathways are essential to form mature biofilms in *M. avium* remain unclear. The characterization of bacterial physiology in biofilms can help in understanding of tolerance mechanisms of the pathogen to antibiotics.

**Figure 4:**
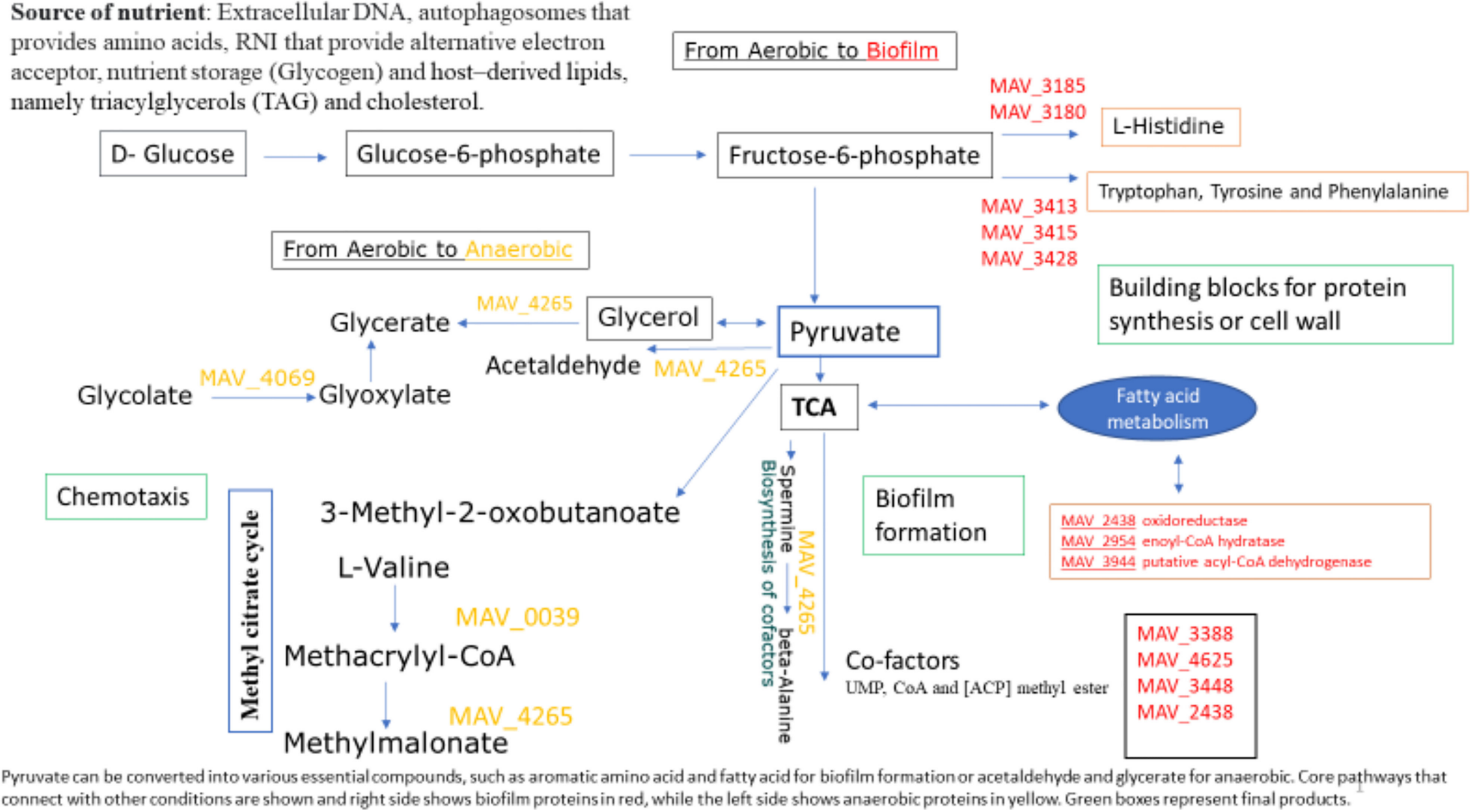
Pathways that remain the same in all environmental conditions. Pyruvate can be converted into various essential compounds, such as aromatic amino acid and fatty acid for biofilm formation or acetaldehyde and glycerate for anaerobic. Core pathways that connect with other conditions are shown and right side shows biofilm proteins in red, while the left side shows anaerobic proteins in yellow. Green boxes represent final products. The mentioned pathways were determined from the Kyoto Encyclopedia of Genes and Genomes (KEGG) databases. The levels of each enzymes within these pathways were quantitated as described in “Methods” section and that did not show significant changes across the different environmental conditions. The level depicted for each enzyme from three independent biological aerobic, anaerobic and biofilms grown for 24 h. Statistical significance was determined using a two-tailed unpaired Student’s t test (p < 0.05).

The analysis of *M. avium* proteome in biofilm and aerobic conditions revealed the metabolic pathways implicated in fatty acid metabolism, and biosynthesis of amino acid and cofactors (**Figure 4**). Numerous proteins involved in the aromatic amino acid (AAA) metabolism were detected in the biofilm condition as well (**Figure 4**). Synthesis of MAV_0344, MAV_3180, MAV_3185, MAV_3413, MAV_3415, MAV_3428 proteins involved in the biosynthesis of amino acid pathway were observed during biofilm formation of *M. avium*. The majority of enzymes of the aromatic amino acid biosynthesis pathway are highly conserved across mycobacterial species with the exception of *M. leprae* (Pradeep et al., 2006). Tryptophan is a member of aromatic amino acid group that has been linked to various metabolic functions involved in the maintenance of redox homeostasis under conditions of reduced oxygen availability and NAD+ biosynthesis (Roager and Licht, 2018). In mature biofilms of *S. Typhimurium*, tryptophan biosynthesis and transport pathways were highly upregulated for bacterial attachment (Hamilton et al., 2009). Moreover, deletion of the *trpE* gene has led to decreased bacterial attachment and a biofilm weakness (Hamilton et al., 2009). Tyrosine, phenylalanine and tryptophan are three aromatic amino acids that are synthesized from the common precursor metabolite chorismate, which originates from the shikimate pathway (Elsden et al., 1976; Entus et al., 2002). The AAA are linked to the synthesis of a variety of secondary metabolites, protein synthesis and anabolic pathways (Ratledge, 1982). In *M. tuberculosis*, the shikimate pathway is essential for viability because it leads to the biosynthesis of a wide range of primary and secondary metabolites, including aromatic amino acids, folate, naphthoquinones, menaquinone (vitamin K2) that is important for the switch between aerobic respiration and anaerobic lactic acid fermentation and in synthesis of mycobactins (Ratledge, 1982; Parish and Stoker, 2002; Smith et al., 2021). The *aroK* gene encodes shikimate kinase, which catalyzes the fifth step in chorismate biosynthesis (Parish and Stoker, 2002). Disruption of *aroK* compromises the *M. tuberculosis* viability even in the presence of exogenous supplementation, suggesting that this pathway is a possible target for anti-mycobacterial agents (Parish and Stoker, 2002; Ely et al., 2008). Additionally, chorismate pathway is absent in the human host, thus targeting chorismate pathway or the mycobacterial enzyme (AroK) is unlikely to have a deleterious side effect on the host (Parish and Stoker, 2002). While amino acid synthesis is essential for protein production, it is important to note that the upregulation in the enzyme level does not necessarily mean an upregulation in the enzyme product as well as or the metabolite. For example, the glycerol-derived metabolites (2-Phosphoglyceric acid, Glycerol 3-phosphate and D-Glyceraldehyde 3-phosphate) were significantly up-regulated during *E. coli*, UTI89 strain biofilm formation, while the concentration of glycerol was decreased considerably (Lu et al., 2019).

Alterations of fatty acid and phospholipid metabolism have been found to be associated with the formation of biofilms (Yao and Rock, 2017). In our study, we identified upregulation of MAV_2438 oxidoreductase, short chain dehydrogenase/reductase family protein, MAV_2945 enoyl-CoA hydratase/isomerase family protein and MAV_3944 putative acyl-CoA dehydrogenase of fatty acid metabolism during the formation of *M. avium* biofilm, all genes observed to be upregulated upon *M.avium* biofilm formation, possibly indicating an association between fatty acid oxidation and biofilm formation. Interestingly, fatty acid metabolism leads to acetyl co-A, and in the presence of MAV_0344 citrate synthase, acetyl-coA is converted to citrate for entry into the TCA cycle (**Figure 4**). It has been previously described that *M. tb* prefers to metabolize fatty acids rather than glucose during persistent infection (Bloch and Segal, 1956). That finding agrees with the fact that the persistence state requires a suitable carbon and energy source (other than glucose) to maintain a low flux status through core metabolic pathways (Amato et al., 2014; Cabral et al., 2018). Furthermore, we have previously observed that the utilization of both short-chain fatty acid (SCFA) and glycerol by both planktonic and biofilm bacteria affects the susceptibility of *M. avium* to clinically used antibiotics in established biofilms (Silva et al., 2020). The results of the study demonstrate that glycerol and the SCFA are processed by *M. avium* in biofilms as an energy source, supporting *M. avium* growth in both biofilms but also in planktonic state (Silva et al., 2020). *M. tb* metabolism is also enhanced, in particular, by a high number of fatty acid beta-oxidation enzymes (Pradeep et al., 2006). In fact, *Mtb* preferentially metabolizes host–derived lipids, namely triacylglycerols (TAG) and cholesterol, in order to maintain virulence (VanderVen et al., 2015). Because glycerol is a host derived carbon source that *M. avium* can utilized at different stages (Silva et al., 2020) and that both *M. avium* planktonic and biofilm phenotypes encounter during the infection, we hypothesized that the fatty acid metabolic pathway is transitional pathway utilized at aerobic and biofilm conditions and can be regarded as novel target for the development of treatments against the *M. avium*.

A number of genes have been identified previously as being involved in the production of *M. avium* biofilms. *guaB2, ccsA, accD2, pks10, pmmB, accA2 and gtf are M. avium* genes up regulated upon the formation of a biofilm (Yamazaki et al., 2006). Yamazaki et al. showed that *gtf* (glycosyltransferase) as a gene which encodes enzyme used during the biosynthesis of GPL (glycopeptidolipid), is essential for initial surface attachment during biofilm formation for *M. avium* (Yamazaki et al., 2006). *gtf* showed 3.6-fold increase upon incubation on polyvinyl chloride (PVC, as *M. avium* is commonly isolated from pvc water pipes) plates using the green fluorescent protein (GFP) promoter library (Yamazaki et al., 2006). Additionally, genetic analysis of *M. smegmatis* demonstrated that genes encode enzymes involved in glycopeptidolipid (GPL) biosynthesis are important for biofilm formation (Carter et al., 2003). In agreement with that we found in our proteomic data, that MAV_1519 glycosyltransferase is also significantly upregulated during biofilm formation for *M. avium* and the strong induction of GPL at both transcription and translation level under biofilm condition confirms its importance for *M. avium* biofilm. Evidence for genes that are expressed specifically in biofilms and therefore mediate biofilm-associated recalcitrance to antibiotics are described in *P. aeruginosa* (Ciofu and Tolker-Nielsen, 2019) and *M. tuberculosis* (Richards et al., 2019). Richards et al. have established a link between the lipopeptide (INLP) as a secondary metabolite essential for *M. tuberculosis* biofilm development and antibiotics tolerance (Richards et al., 2019).

### Identification of biofilm associated transporters in *M. avium*

In most cases, the bacterial metabolism of nutritional compounds starts with their transport across the cell membrane mediated by a specific transport system (Kleiner, 1985). Bacterial transport systems enable bacteria to accumulate needed nutrients and remove waste products, thus allowing bacteria to grow and to survive stress conditions (Padan, 2009). Under biofilm conditions MAV_2903 putative Mg_2_+ transporter-C (MgtC) and MAV_3775 ammonium transporter was observed being upregulated in *M. avium*. Also, this study identified nitrate, nitrite transporter and nitrite reductase enzymes of *M. avium* highly upregulated under anaerobic and biofilm conditions. In *M. tuberculosis*, it has been shown that an increased in the amount of nitrate can be used to support bacterial survival during hypoxia by replacing oxygen as terminal electron acceptor (Khan and Sarkar, 2012). Nitrogen metabolism and alternative nitrogen sources such as ammonium have been reported in Gram-positive bacteria as well (Merrick and Edwards, 1995). Ammonium transport linked to nitrogen uptake is regulated *via* AmtB, a well-conserved ammonium transport membrane protein present in many bacterial species (Arai, 2011). In *Streptococcus mutans* the ammonium transporter, *nrgA*, is required for the transport and utilization of ammonium and for bacteria growth as well (Ardin et al., 2014). In the presence of 20 mM ammonium chloride, the growth of *nrgA*-deficient mutant strain (NRGD) was clearly delayed as compared to that of the wild type at pH 5.0, but slightly changed as compared to that of the wild-type at pH 7.0. Suggesting that the ammonium transporter may be sensitive to acidic conditions (Ardin et al., 2014). NRGD developed a reduced biofilm mass in comparison to the wild type (Ardin et al., 2014). These results suggest that the *nrgA* gene in *S. mutans* is essential for export of molecules, growth and biofilm formation (Ardin et al., 2014). MAV_3775 ammonium transporter is not well characterized; however, it is expected to observe upregulation in ammonia levels during the stages of biofilm formation because the amino acids were metabolized under biofilm conditions (**Figure 4**). While we do not have a time point measurement of the ammonium level during the stages of biofilm formation, we expect that ammonia will continue to accumulate over time because of its significance in hypoxic phases as well. In *B. pseudomallei*, at 2 weeks, ammonia levels reached a maximum concentration of 7 mM suggesting that amino acids were metabolized anaerobically, resulting in ammonia accumulation in the spent medium (Hamad et al., 2011).

Since *mgtC* is part of an operon with *mgtB* which encodes a Mg_2_+-transporting P-type ATPase, MgtC was hypothesized to function in ion transport, possibly in Mg_2_+ transport (Günzel et al., 2006). The mgtC locus is regulated by the PhoP/PhoQ two-component system, a two-component system that governs virulence functions, mediates the adaptation to Mg_2_+-limiting environments, and present in both pathogenic and non-pathogenic bacterial species (Blanc-Potard and Groisman, 1997; Le Moigne et al., 2016). In *Salmonella typhimurium* MgtC protein may be involved in regulating membrane potential but does not directly transport Mg2+ or another ion (National Center for Biotechnology Information, 2022). Also, it is necessary for Salmonella virulence, and normal growth in low Mg2+ which is a mechanism responsible for the expression of pathogenicity islands in enteric bacteria (Bhusal et al., 2017). A serovar Typhimurium strain lacking *mgtC* exhibits significant attenuation in a mouse model of infection after intraperitoneal injection (Bhusal et al., 2017). In *Mycobacteriodes abscessus*, MAB_3593 encodes MgtC, a known virulence factor which has a role when bacteria reside inside macrophages and during adaptation to Mg ++ deprivation (Wayne and Hayes, 1996). However, *M. abscessus* knock-out *mgtC* mutant growth in macrophages (J774 or THP1 cells) was not affected (Wayne and Hayes, 1996). Additionally, our laboratory has identified that the presence of Mg ++ in the surrounding environment stimulates *M. abscessus* biofilm formation (Gould et al., 2006). However, the role of Mg ++ or the MAV_2903 putative Mg2+ transporter-C (MgtC) for *M. avium* biofilm is presently unknown. Because of the fact that MgtC contributes to intramacrophage survival of numerous pathogens as well as to bacterial adaptation in environments with limited Mg++ concentrations and its overexpression in our proteomics data under biofilm conditions, it will be interesting to evaluate its role during *M. avium* infection.

### Common enzymes in anaerobic and biofilm associated stress conditions that are involved in metabolic rewiring of *M. avium*

**Figure 5:**
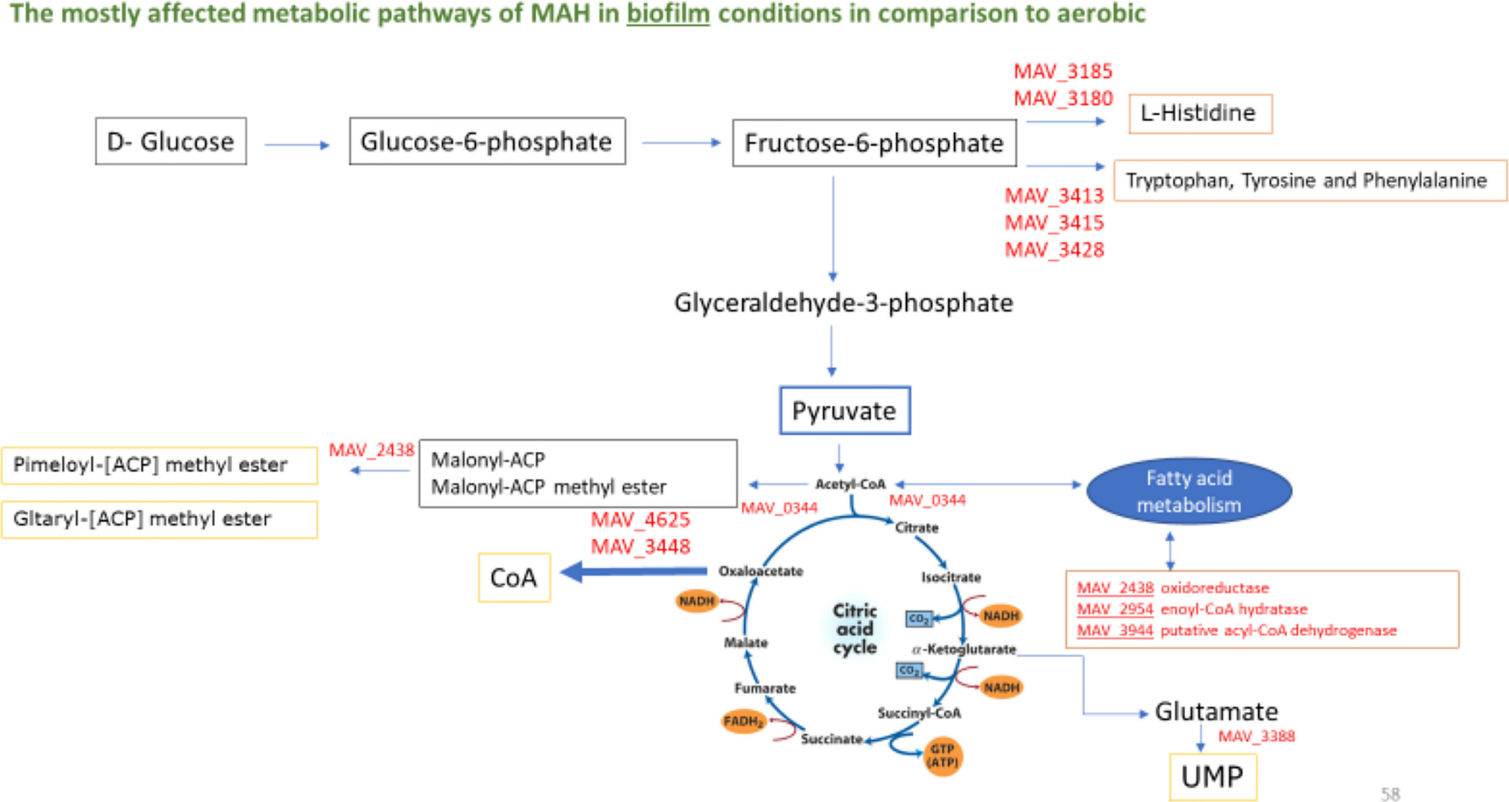
Biosynthesis of cofactors (Yellow Boxes), fatty acid metabolism (Brown Box) and amino acid metabolism (Orange Boxes) and entry into the TCA cycle during biofilm growth of M. avium. The represented enzymes were synthesized ≥1.5-fold over aerobic control. Fatty acid metabolism (MAV_2438 oxidoreductase, short chain dehydrogenase/reductase family protein, MAV_2945 enoyl-CoA hydratase/isomerase family protein, MAV_3944 putative acyl-CoA dehydrogenase).Biosynthesis of amino acids (MAV_0344 citrate synthase 2, MAV_3180 phosphoribosyl-AMP cyclohydrolase, MAV_3185 hisB; imidazoleglycerol-phosphate dehydratase, MAV_3413 aroQ; 3-dehydroquinate dehydratase, type II, MAV_3415 aroB; 3-dehydroquinate synthase, MAV_3428 aroE; shikimate-5-dehydrogenase). Biosynthesis of cofactors (MAV_0995 moaE; molybdopterin converting factor, subunit 2, MAV_2438 oxidoreductase, short chain dehydrogenase/reductase family protein, MAV_3388 pyrF; orotidine 5’-phosphate decarboxylase, MAV_3448 2-dehydropantoate 2-reductase, MAV_4265 aldehyde dehydrogenase (NAD) family protein, MAV_4625 panE; 2-dehydropantoate 2-reductase, MAV_4748 thiE; thiamine-phosphate pyrophosphorylase).

Proteome analysis of *M. avium* cells during exposure to aerobic and biofilm conditions revealed a set of shared enzymes that could be associated with bacterial tolerance. **Figure 5** points to the overproduction of MAV_0357 haloalkane dehalogenase, MAV_0039 putative acyl-CoA dehydrogenase, MAV_4265 aldehyde dehydrogenase (NAD) family protein, MAV_4069 KatE catalase HPII and MAV_0927 conserved hypothetical protein. The upregulation of those proteins supports the involvement of metabolic pathways of chloroalkane degradation, glycerolipid metabolism, fatty acid metabolism, degradation of aromatic compounds and glyoxylate cycle. Enzymes involved in the chloroalkane and chloroalkane degradation pathway were in greater abundance at both stress conditions, suggesting its essentiality for *M. avium* infection. Acetaldehyde is the product of chloroalkane pathway which reversibly can be converted into an acetyl-CoA, acetyl-phosphate and pyruvate, and then processed in the TCA cycle for ATP synthesis (Rojony et al., 2019). On the other hand, aerobic bacteria metabolized acetaldehyde to produce acetate in the presence of aldehyde dehydrogenase (Nosova et al., 1996). Acetate is a short chain fatty acid that could have diverse advantages to *M. avium* infection in anaerobic conditions suggesting that chloroalkane degradation pathway could be used to transition from aerobic to anaerobic conditions. *M. avium* central metabolism leads to the production of acetoin under aerobic conditions and lactic acid mainly under anaerobic conditions (Pradeep et al., 2006). lactic acid and acetoin are results of bacterial metabolism anaerobically and aerobically, respectively (Pradeep et al., 2006). Because the chloroalkane pathway of *M. avium* was enriched significantly under anaerobic stress, it is predicted to participate in *M. avium* anaerobic metabolism. Therefore, the production of acetate from the chloroalkane pathway could indicate *M. avium* strategy to occupy all the binding sites available for the enzymes that catalyze the production of acetoin from pyruvate, thus limiting its production and alternatively supporting the anaerobic fermentation over the aerobic respiration. This phenomenon clearly occurs during macrophage infection by Mtb (Somashekar et al., 2011). It has been reported that host‐derived lactate also supports *Neisseria meningitidis* (Exley et al., 2005) and *Salmonella enterica* (Gillis et al., 2018) virulence. Chloroalkene degradation also produces substrate for the glyoxylate shunt, which is a modified Krebs cycle that occurs in mycobacteria (Serafini et al., 2019). The glyoxylate cycle is comprised of many of the same reactions as the TCA cycle, but it does not include the two decarboxylation reactions (National Center for Biotechnology Information, 2022). To cope with metabolic‐challenging environments, Mtb uses glyoxylate shunt and reverse methylcitrate cycle to allow optimal metabolism of lactate and pyruvate (Serafini et al., 2019). Additionally, glyoxylate cycle and, therefore, enzymes associated with the glyoxylate shunt is essential for the persistence and virulence of Mtb (Gould et al., 2006). Because the isocitrate lyase (ICL) converts the isocitrate into glyoxylate and malate synthase, which in turn catalyzes the conversion of malate from glyoxylate, ICL has been proposed for the development of additional anti-TB therapy (Bhusal et al., 2017). In addition, reductive amination of the glyoxylate by glycine dehydrogenase has been demonstrated to be an alternative energy source for *M. tuberculosis* during nonreplicative persistence state and aids the pathogen in surviving anaerobic conditions (Wayne and Hayes, 1996). Another pathway upregulated in MAH under the anaerobic and biofilm conditions is degradation of aromatic compounds. As shown in **Figure 5**, the trans-cinnamate and phenylpropanoate are used to form fumarate and succinate by MAV_0927 conserved hypothetical protein. The effects of fumarate supplementation on transition from anaerobic to aerobic growth *in vitro* as well as inside macrophages was confirmed in *L. monocytogenes* (Wallace et al., 2018).

### Effect of fatty-acid synthesis inhibitors

Although the concentration of the utilized compounds need to kill *M.avium* are beyond of the achievable serum level, those compounds can be used experimentally to inhibit pathways. It was decided that 50% the MIC did not show any significant effect of bacterial growth (data not shown). Brief incubations of 5 minutes or 30 minutes were performed prior to expose the bacteria to a surface. As shown in **Table 1**, both compounds at sub-inhibitory concentration had a significant effect on biofilm formation, the first step used by the pathogen to establish a lung infection.

**Table 1:** Effect of Isoniazid and Triclosan on the transition between aerobic growth and biofilm.

Conditions	CFU/ml		Biomass ^1^
Day	0	7	7
Initial inoculum	3 x 10^8^		
Control 5 min	3.6 x 10^7^	7.1 + 0.4 x 10^7^	2.369 + 0.122
Control 30 min	3.8 x 10^7^	7.5 + 0.3 x 10^7^	2.386 + 0.141
Isoniazid 5 min		2.1 + 0.5 x 10^6*^	1.214* + 0.068
Isoniazid 30 min		5.8.+ 0.4 x 10^4**^	0.377** + 0.045
Triclosan 5 min		3.5 + 0.3 x 10^6^ *	1.321* + 0.091
Triclosan 30 min		7.2 + 0.5 x 10^4^**	0.858**+ 0.837

*P < 0.05 compared to control.

**P < 0.001 compared to control.

Experiments were repeated 3 times. The values represent mean + SD. ANOVA was used to calculate the significance of the differences.

1. Biomass determined after staining with crystal violet as described in references 29, 58.

### Regulatory systems

TetR/AcrR family proteins act as global multi-target regulators. It regulates a wide range of cellular activities, such as osmotic stress, homeostasis, biosynthesis of antibiotics, multidrug resistance, efflux pumps, enzymes implicated in different catabolic pathways, virulence and pathogenicity of bacteria (Cuthbertson and Nodwell, 2013). The molecular function of TetR/AcrR family repressors is based on the binding to incomplete palindromic sequences in the upstream region of their own gene or an intergenic region between the repressor and regulated genes (Cuthbertson and Nodwell, 2013). The first member of TetR was identified in *Escherichia coli* and controls the expression of the gene encoding a tetracycline efflux pump responsible for drug resistance conferred by Tn10 (Lin et al., 2005). Because TetR/AcrR family proteins function as efflux pumps and transporters involved in antibiotic resistance and tolerance to toxic chemical compounds (Lin et al., 2005), it is considered a broad-spectrum drug target (Colclough et al., 2019).

**Figure 6:**
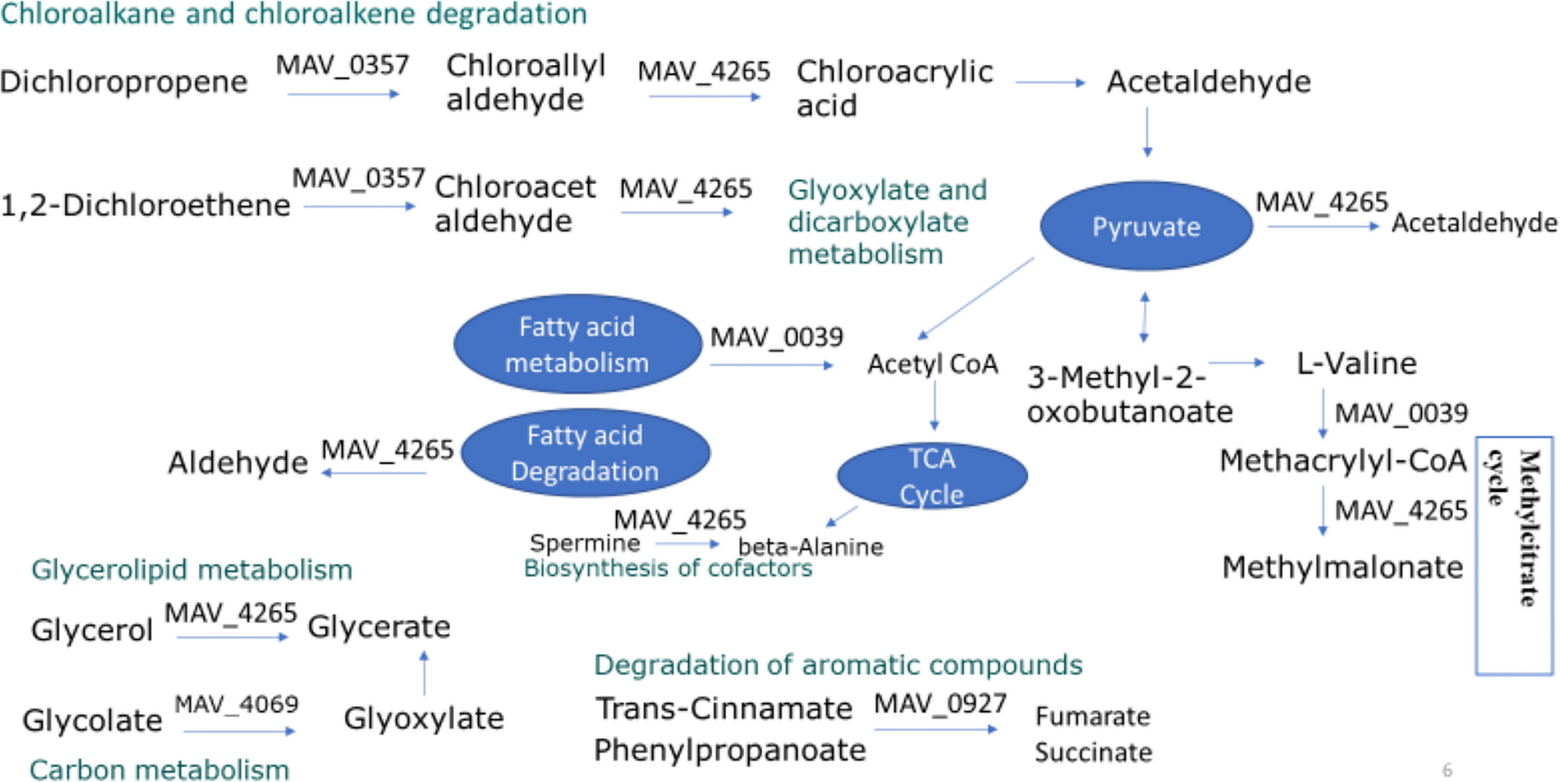
Upregulated metabolic pathways of *M. avium* in anaerobic and biofilm conditions. Synthesized proteins identified during different growth conditions (anaerobic and biofilm) was compared with the levels of corresponding protein found in standard growth condition (aerobic). The induction reaching 1.5-fold or higher in comparison to aerobic are presented. MAV_0357, haloalkane dehalogenase, MAV_0039, putative acyl-CoA dehydrogenase, MAV_4265, aldehyde dehydrogenase (NAD) family protein, MAV_4069 KatE catalase HPII, MAV_0927 conserved hypothetical protein.

In our data, a TetR-family regulator, MAV_5151 was found to regulate several virulence traits by responding to fluctuating environmental nutrients and oxygen levels in the surroundings. MAV_5151 repress the expression of several proteins known to be associated with oxidative stress such as oxidoreductase (**Figure 6**). MAV_5153 starvation-inducible DNA-binding protein and MAV_5152 oxidoreductase are both repressed by MAV_5151. It seems that MAV_5151 repressing the oxidative stress proteins during adverse environmental challenges, to efficiently utilize the nutrients and energy sources in the activation of virulence traits. It will be interesting to create GFP reporter assays to examine the examine the expression of MAV_5152 and MAV_5153 during the transition to post exponential growth phase in a mutant that lacks MAV_5151 to confirm its repressing activity.

Thus far, our current understanding of TetR/AcrR is largely based on those we have characterized well and for which we have crystal structures. The crystal structure of TetR from *Mycobacterium smegmatis* revealed that both dimeric Ms6564 molecules were found to bind to opposite sides of their cognate DNA (Yang et al., 2013) Eventually, that allow a sliding motion of the regulator along the genome DNA and extensively regulate the expression of diverse genes in *M. smegmatis* (Yang et al., 2013). It will be interesting to further examine the operator recognition mechanism of MAV_5151 in association with other TetR family proteins to elucidate its potential as a drug target.

The Cyclic AMP receptor proteins, Crp and Fnr (fumarate and nitrate reductase) are global transcription regulators that control cell functions and signaling pathways (Galimand et al., 1991). Crp/Fnr family control responses to a variety of stress signals, such as anoxia, temperature, and oxidative and nitrosative stress (Mesa et al., 2006). The characteristic structure of Crp/Fnr is a Cyclic nucleotide-binding (121 amino acid in length) and C-terminal helix-turn-helix (HTH) motif (74 amino acid in length) that have a molecular function of DNA binding. In our data MAV_0092 was upregulated in both biofilm and anaerobic conditions suggesting its role in environmental adaptation, such as O_2_ limiting conditions. MAV_0092 assignment to KEGG (Kyoto Encyclopedia of Genes and Genomes) metabolic pathways reveals its involvement in two pathways; two component systems and quorum sensing. Fnr is the master regulator responsible for the metabolic transition between aerobic and anaerobic growth by directly activating operons encoding nitrate/nitrite reductases (Lamberg and Kiley, 2000; Crack et al., 2004). Nitrogen metabolism under anaerobic and biofilm conditions promotes the pathogen virulence and tolerance under hypoxic stress. For example, in the absence of oxygen, *Pseudomonas aeruginosa* uses nitrate or nitrite for respiration (Schobert and Jahn, 2010). Although little is known about the regulation of nitrate and nitrite respiration in *M. avium*, it will be interesting to examine knockout mutants for Crp/Fnr regulator and functionally characterized anaerobic respiration of nitrate and fumarate under the direct control of two component systems.

It has been reported that Crp/Fnr transcription factor Lmo0753, is necessary for biofilm formation in *L. monocytogenes* (Salazar et al., 2013). Because of the amino acid sequence similarity between Lmo0753 and MAV_0092, we hypothesize that MAV_0092 may also play a role in environmental persistence-related mechanisms in *M. avium* such as biofilm formation. Additionally, Crp regulates genes for quorum sensing and biofilm formation. For example, Crp in *Yersinia pestis*, forms biofilm *via* different mechanisms, such as RNA-binding regulatory protein CsrA (Willias et al., 2015) and Crp can directly binds to the promoter of the acyl-homoserine lactone (AHL, quorum sensing genes) receptor, *ypeR*, and thereby controls efficient production of AHLs within biofilms (Itzert et al., 2019).

## Conclusions

In summary, this study identified the metabolic pathways, transporters and transcription regulators with the intent to better understand *M. avium* adaptation to environmental stress conditions in the airway. By combining proteomics and metabolic pathways, the study has revealed that obviously metabolic reprogramming triggered biofilm formation and anaerobic survival compared to the aerobic population. As we found a large number of small-molecule metabolites and co-factors are essential for biofilm formation, those differential metabolites and the associated metabolic pathways can be regarded as novel targets for the development of biofilm-based treatments and antibiotic discovery against *M. avium* infection. More importantly, such effort could provide a novel insight into better understanding the reprogramming of metabolism during adaptation of *M. avium* to stressful environments such as nutrient deprivation and low oxygen for long-term survival during disease. Delineation of the metabolism of *M. avium* during the disease progression will provide new avenues for the development of advanced and antibiotic sparing approaches to the prevention and treatment of *M. avium* infection.

## Data availability statement

The datasets presented in this study can be found in online repositories. The names of the repository/repositories and accession number(s) can be found in the article/**Supplementary Material**.

## Author contributions

NA, performed the study, analyzed the data wrote the paper. RR, Performed the study and help with the analysis of the data. LD, Analyzed the data, edited the manuscript. LB, Designed the study, analyzed the data, edited the manuscript and funded the work. All authors contributed to the article and approved the submitted version.

## Supplementary material

Supplemental 1 Proteins downregulated and proteins upregulated.

Supplemental 2 Proteins Upregulated in different conditions.
