# Supplementary material for: Metabolic pathways that permit Mycobacterium avium subsp. hominissuis to transition to different environments encountered within the host during infection
Source: Front Cell Infect Microbiol. 2023 Apr 14;13:1092317. doi: 10.3389/fcimb.2023.1092317 (PMC10140322; doi:10.3389/fcimb.2023.1092317)
Supplement: Supplementary file 3 [file Table_3.docx]

**Supplementary # 3**

Repressed enzymes by ≤ 0.5-fold lower than control in anaerobic and biofilm stress conditions

| **Common metabolic pathways and associated enzymes** |
| --- |
| - ABC transporters - Mycobacterium avium 104 ([48](javascript:display('mav02010'))) - [mav:MAV_0238](https://www.genome.jp/entry/mav:MAV_0238) O-antigen export system ATP-binding protein RfbB - [mav:MAV_0317](https://www.genome.jp/entry/mav:MAV_0317) ABC transporter, quaternary amine uptake transporter (QAT) family protein, substrate-binding protein - [mav:MAV_0318](https://www.genome.jp/entry/mav:MAV_0318) amino acid ABC transporter, ATP-binding protein - [mav:MAV_0319](https://www.genome.jp/entry/mav:MAV_0319) ABC transporter, permease protein - [mav:MAV_0320](https://www.genome.jp/entry/mav:MAV_0320) ABC transporter, permease protein - [mav:MAV_0464](https://www.genome.jp/entry/mav:MAV_0464) bacterial extracellular solute-binding protein, family protein 5 - [mav:MAV_0465](https://www.genome.jp/entry/mav:MAV_0465) dppB; ABC transporter, permease protein DppB - [mav:MAV_0466](https://www.genome.jp/entry/mav:MAV_0466) dppC; ABC transporter, permease protein DppC - [mav:MAV_0467](https://www.genome.jp/entry/mav:MAV_0467) dppD; ABC transporter, ATP-binding protein DppD - [mav:MAV_0767](https://www.genome.jp/entry/mav:MAV_0767) pstB; phosphate ABC transporter, ATP-binding protein PstB - [mav:MAV_0946](https://www.genome.jp/entry/mav:MAV_0946) TrnB1 protein - [mav:MAV_0947](https://www.genome.jp/entry/mav:MAV_0947) TrnB2 protein - [mav:MAV_0950](https://www.genome.jp/entry/mav:MAV_0950) virulence factor Mce family protein - [mav:MAV_0951](https://www.genome.jp/entry/mav:MAV_0951) virulence factor mce family protein - [mav:MAV_1047](https://www.genome.jp/entry/mav:MAV_1047) pstS; phosphate ABC transporter, phosphate-binding protein PstS - [mav:MAV_1048](https://www.genome.jp/entry/mav:MAV_1048) pstC; phosphate ABC transporter, permease protein PstC - [mav:MAV_1049](https://www.genome.jp/entry/mav:MAV_1049) pstA; phosphate ABC transporter, permease protein PstA - [mav:MAV_1271](https://www.genome.jp/entry/mav:MAV_1271) conserved hypothetical protein - [mav:MAV_1374](https://www.genome.jp/entry/mav:MAV_1374) Bacterial extracellular solute-binding protein - [mav:MAV_1375](https://www.genome.jp/entry/mav:MAV_1375) sugA; ABC transporter, permease protein SugA - [mav:MAV_1376](https://www.genome.jp/entry/mav:MAV_1376) sugB; ABC transporter, permease protein SugB - [mav:MAV_1377](https://www.genome.jp/entry/mav:MAV_1377) sugC; ABC transporter, ATP-binding protein SugC - [mav:MAV_1421](https://www.genome.jp/entry/mav:MAV_1421) ABC transporter, ATP-binding protein - [mav:MAV_1429](https://www.genome.jp/entry/mav:MAV_1429) bacterial extracellular solute-binding protein, family protein 5 - [mav:MAV_1431](https://www.genome.jp/entry/mav:MAV_1431) oppD; ABC transporter, ATP-binding protein OppD - [mav:MAV_1433](https://www.genome.jp/entry/mav:MAV_1433) oppB; ABC transporter, permease protein OppB - [mav:MAV_1566](https://www.genome.jp/entry/mav:MAV_1566) putative ABC transporter ATP-binding protein - [mav:MAV_1567](https://www.genome.jp/entry/mav:MAV_1567) ABC transporter, ATP-binding protein - [mav:MAV_1782](https://www.genome.jp/entry/mav:MAV_1782) sulfate ABC transporter, sulfate-binding protein - [mav:MAV_1783](https://www.genome.jp/entry/mav:MAV_1783) cysT; sulfate ABC transporter, permease protein CysT - [mav:MAV_1784](https://www.genome.jp/entry/mav:MAV_1784) cysW; sulfate ABC transporter, permease protein CysW - [mav:MAV_1785](https://www.genome.jp/entry/mav:MAV_1785) sulfate/thiosulfate import ATP-binding protein CysA - [mav:MAV_2083](https://www.genome.jp/entry/mav:MAV_2083) cobalt transport protein - [mav:MAV_2435](https://www.genome.jp/entry/mav:MAV_2435) sulfonate binding protein - [mav:MAV_2533](https://www.genome.jp/entry/mav:MAV_2533) virulence factor Mce family protein - [mav:MAV_2860](https://www.genome.jp/entry/mav:MAV_2860) molybdenum import ATP-binding protein ModC - [mav:MAV_2861](https://www.genome.jp/entry/mav:MAV_2861) modB; molybdate ABC transporter, permease protein - [mav:MAV_2863](https://www.genome.jp/entry/mav:MAV_2863) modA; molybdate ABC transporter, periplasmic molybdate-binding protein - [mav:MAV_2896](https://www.genome.jp/entry/mav:MAV_2896) SbmA protein - [mav:MAV_3400](https://www.genome.jp/entry/mav:MAV_3400) ABC transporter substrate-binding protein - [mav:MAV_4002](https://www.genome.jp/entry/mav:MAV_4002) efflux ABC transporter, permease protein - [mav:MAV_4003](https://www.genome.jp/entry/mav:MAV_4003) ftsE; cell division ATP-binding protein FtsE - [mav:MAV_4239](https://www.genome.jp/entry/mav:MAV_4239) pstS; phosphate ABC transporter, phosphate-binding protein PstS - [mav:MAV_4504](https://www.genome.jp/entry/mav:MAV_4504) ABC transporter, ATP-binding protein - [mav:MAV_4553](https://www.genome.jp/entry/mav:MAV_4553) conserved hypothetical protein - [mav:MAV_5016](https://www.genome.jp/entry/mav:MAV_5016) conserved hypothetical protein - [mav:MAV_5017](https://www.genome.jp/entry/mav:MAV_5017) conserved hypothetical protein - [mav:MAV_5100](https://www.genome.jp/entry/mav:MAV_5100) ABC transporter, ATP-binding protein |
| - Glycerolipid metabolism - Mycobacterium avium 104 ([25](javascript:display('mav00561'))) - [mav:MAV_0214](https://www.genome.jp/entry/mav:MAV_0214) antigen 85-A - [mav:MAV_0355](https://www.genome.jp/entry/mav:MAV_0355) bifunctional wax ester synthase/acyl-CoAdiacylglycerol acyltransferase - [mav:MAV_0406](https://www.genome.jp/entry/mav:MAV_0406) glpK; glycerol kinase - [mav:MAV_0713](https://www.genome.jp/entry/mav:MAV_0713) aldehyde dehydrogenase (NAD) family protein - [mav:MAV_1275](https://www.genome.jp/entry/mav:MAV_1275) aldehyde dehydrogenase family protein - [mav:MAV_1340](https://www.genome.jp/entry/mav:MAV_1340) aldehyde dehydrogenase - [mav:MAV_1643](https://www.genome.jp/entry/mav:MAV_1643) aldehyde dehydrogenase - [mav:MAV_1686](https://www.genome.jp/entry/mav:MAV_1686) acyltransferase, ws/dgat/mgat subfamily protein - [mav:MAV_1825](https://www.genome.jp/entry/mav:MAV_1825) aldehyde dehydrogenase family protein - [mav:MAV_1847](https://www.genome.jp/entry/mav:MAV_1847) acyltransferase, ws/dgat/mgat subfamily protein - [mav:MAV_1922](https://www.genome.jp/entry/mav:MAV_1922) aldehyde dehydrogenase - [mav:MAV_2184](https://www.genome.jp/entry/mav:MAV_2184) conserved hypothetical protein - [mav:MAV_2240](https://www.genome.jp/entry/mav:MAV_2240) acyltransferase, ws/dgat/mgat subfamily protein - [mav:MAV_2313](https://www.genome.jp/entry/mav:MAV_2313) 1-acylglycerol-3-phosphate O-acyltransferase - [mav:MAV_2647](https://www.genome.jp/entry/mav:MAV_2647) betaine-aldehyde dehydrogenase - [mav:MAV_2816](https://www.genome.jp/entry/mav:MAV_2816) antigen 85-B - [mav:MAV_3079](https://www.genome.jp/entry/mav:MAV_3079) HAD-superfamily protein hydrolase, subfamily protein IIA - [mav:MAV_3220](https://www.genome.jp/entry/mav:MAV_3220) glycerol-3-phosphate acyltransferase - [mav:MAV_3352](https://www.genome.jp/entry/mav:MAV_3352) acyltransferase, ws/dgat/mgat subfamily protein - [mav:MAV_4196](https://www.genome.jp/entry/mav:MAV_4196) acyltransferase, ws/dgat/mgat subfamily protein - [mav:MAV_4265](https://www.genome.jp/entry/mav:MAV_4265) aldehyde dehydrogenase (NAD) family protein - [mav:MAV_4952](https://www.genome.jp/entry/mav:MAV_4952) acyltransferase, ws/dgat/mgat subfamily protein - [mav:MAV_5147](https://www.genome.jp/entry/mav:MAV_5147) fatty aldehyde dehydrogenase - [mav:MAV_5183](https://www.genome.jp/entry/mav:MAV_5183) antigen 85-C - [mav:MAV_5202](https://www.genome.jp/entry/mav:MAV_5202) aldehyde dehydrogenase |
